# 953. Containing the Problem: Utilizing Quality Improvement Process to Enhance Fecal Management System-related Outcomes

**DOI:** 10.1093/jbcr/irag033.520

**Published:** 2026-04-06

**Authors:** Shawna Hardoby, Carey K Lamphier, Abby L Hrabovsky, Laura S Johnson, Julie A Childers, Lauren B Nosanov

**Affiliations:** Grady Health System, Atlanta, GA; Grady Memorial Hospital, Atlanta, GA; Walter L. Ingram Burn Center at Grady Memorial Hospital, Atlanta, GA; Grady Memorial Hospital / Emory School of Medicine / Morehouse School of Medicine, Atlanta, GA; Grady Memorial Hospital, Atlanta, GA; Emory University School of Medicine, Atlanta, GA

## Abstract

**Introduction:**

Patients with extensive burns or compromised skin integrity are highly susceptible to complications from fecal contamination. Prevention of wound soiling is essential to reduce infection risk, protect fragile tissue, and improve comfort. Fecal Management Systems (FMS) divert stool, minimize skin breakdown, and decrease incontinence-related complications and costs. FMS use carries risks, including stool leakage, diminished anal sphincter tone, infection, rectal trauma, bowel obstruction or perforation, and pressure necrosis. At our burn center, two patients experienced severe rectal injury requiring intervention after FMS placement, prompting an in-depth review of practice.

**Methods:**

Index cases were evaluated through the burn program’s Performance Improvement process. Acute Cause Analysis was performed with nursing leadership, informatics, and system quality teams, identifying opportunities with electronic medical record (EMR) order sets, nurse flowsheet documentation, and staff education. Beginning August 2024, real-time audits of all patients with an indwelling FMS were implemented, and charge nurses and nurse managers assessed device indication, securement, monitoring, and documentation.

**Results:**

Audits revealed inconsistent documentation, order set variability, and staff knowledge gaps. EMR improvements deployed in September 2024 (flowsheet redesign) and January 2025 (updated FMS order set) improved standardization and auditability. Hands-on training sessions had an 86% completion rate by burn nursing staff. In partnership with the hospital’s Value Analysis team, a newer FMS device designed to reduce rectal trauma was introduced in April 2025. Product trial is ongoing with no reported device-related complications to date and favorable preliminary experience.

**Conclusions:**

Structured real-time audits identified critical gaps in practice, documentation, and system processes surrounding FMS use. System-level interventions improved compliance, workflow clarity, and patient safety.

**Applicability of Research to Practice:**

Burn unit process optimization drives system-wide improvements. In the post-COVID era, variable baseline staff competency in knowledge and skill exists even among experienced clinicians due to high turnover, redeployment, reliance on travel nurses, and disruptions in traditional training during the pandemic. Structured, hands-on education programs can rebuild consistency, reinforce safe practices, and ensure confidence with high-risk devices. Simulation-based learning, embedding competency assessment into daily workflows, bedside competency checks, and real-time audit/feedback loops allow early identification, timely reinforcement and increases exposure to specialized devices. Engaging bedside clinicians in device trials and system-level changes improves adoption, restores professional agency, and ensures safer, effective care for patients.

**Funding for the study:**

N/A.

